# 

**Published:** 1903-10-03

**Authors:** 


					Publications.
G R IFFIN'S STANDARD PUBLICAT IONS.
835" " Dr. Humphry's book is a distinct advance on all previous Manuals."?British Medical Journal.
Twenty-sixth Edition, with numerous Illustrations, Price 3s. 6d., post free.
A MANUAL OF NURSING, Medical and Surgical.
By LAURENCE HUMPHRY, M.A., M.D., M.R.C.S.,
Assistant Physician and formerly Lecturer to Probationers at Addenbrooke's Hospital, Cambridge.
General Contents.?The General Management of the Sick Eoom in Private Houses?General Plan of the Human Body?Diseases of the Nervotu
System?Respiratory System?Heart and Blood Vessels?Digestive System?Skin and Kidneys?Fevers?Diseases of Children?Wounds and their Complica-
tions?Fractures?Operations and Special Surgical Oases?Management of Child-Bed?Appliances?Antiseptic Treatment?Bandaging?Artificial Respiration
?Application of Electricity?Massage?Sick-Room Cookery, &c., &c.
" We should advise all nurses to possess a copy of the work. We can confidently recommend it as an excellent guide and companion."?The Hospital
TTHE WIFE AND MOTHER: A Medical Guide
to the Oare of her Health and the M*nagem?nt of her Children. By
ALBERT WESTLAND, M.A., M.D., C.M. Third Edition. Hand-
some cloth, 5s.
INFANCY AND INFANT REARING. By
JOHN B. HELLI&R. MD, Lecturer on Diseases of Women and
Children. Yorkshire College; Surgeon to the Hospital for Women
and Children, Leeds. Handsome cloth. Illustrated, 3s.
THE DISEASES OF CHILDREN. A Clinical
Handbook. By GEO. ELDEB, M.D., F.R.O.P.Ed? and J. S.
FOWLER, M.B. F.R.O.P.Ed.. Clinical Tutors, Royal Infirmary,
Edinburgh. With Plates and Illustrations. Leather limp, 10s. 6d.
A SURGICAL HANDBOOK. For Practitioners
and Students, House Surgeons, and Dressers. By F. M. CAIRD,
F.R.C.S., and C. W. CATHOART, F.R.C.S., Assistant Surgeons,
Royal Infirmary, Edinburgh. Illustrated. Twelfth Edition, 8s. 6d.
A MEDICAL HANDBOOK, for the Use of Prac-
titioners and Students. By R. S. AITOHISON, M.D. Edin., F.R.C.P.
Thibd Edition. Thoroughly revised and brought up to date. With
numerous Illustrations. 8s. 6d.
THE DISEASES OF WOMEN (Outlines of).
By JOHN PHILLIPS, M.A., M.D., F.R.C.P., Physician, British
Lying-in Hospital, Examiner in Midwifery to the Royal College of
Phjsicians, &c. With Illustrations. Third Edition. Thoroughly
Revised and Enlarged, 7s. 6d.
MIDWIFERY. For the Use of Young Practi-
tioners, Students, and Midwives. By ARCHIBALD DONALD,
M.A., M.D., C.M.Edin., Surgeon to SC. Mary's Hospital for Women
and Children, Manchester. With numerous Illustrations and
Coloured Plate. Fif.th Edition, Revised, 5s.
HYGIENE (A Handbook of). By Lt.-Col. A. M.
DA.VIES, D.P.H Camb., late A.=si=taut Professor of Hygiene, Army
Medical School, and Bacteriologist, Army Headquarters India. Second
Edition, Bevised and Enlarepo. With Illustrations, 8s. 6d. net.
PR ACTIO fi.L SANITATION. By GEO. REID,
M.D.. D P.H.. Medical Officer. Staffs. County Council. With an Ap-
pendix on Sanitary Law by Herbrrt Manley, M.A., D.P.H. Tenth
Edition Illustrated. Handsome cloth, 6s.
CONSUMPTION (The Hygienic Prevention of).
By J. ED. SQUIRE, M.D., D.P.ti.Camb., Physician to the North
London Hospital for Consumption and Diseases of the Chest. With
frontispiece in Colours. 6s.
FOODS AND DIETARIES: When and How to
Feed the Sick. A munual of Clinical Dietetics. By ROBERT W.
BURNET, M.D., P.R.C.P., Senior Physician, Great Northern Central
Hospital. Third Edition. 4'.
METHODS AND CALCULATIONS in PUBLIC
HEALTH AND VITAL STATISTIC?. Per Students preparing for
Examinations in Public Health and Hygiene. By H. W. G. MA.CLEOD,
M.D.. C.M., D.P.H. In crowi 8vo. With Illustrations. [At press.
PRACTICAL PHARMACY AND DISPENS-
ING (Elements of). By W. ELBORNE, B.A., F.L.S., F.C.S.,
Demonstrator of Materia Medica and Teacher of Pharmacy at
University College, London; Pharmacists to University College
HosDltal. 83. 6d.
AMBULANCE (A Manual of). By J. SCOTT
RIDDELL, O.M., M.B., M.A., Assistant Surgeon Aberdeen Royal
Infirmary; Examiner to the St. John, St. Andrews, and Aberdeen
Ambulance Associations. Fourth Edition. With numerous Illus-
trations and fall-page Plates. 4s.
Thirty-Seventh Edition. Royal 8vo. handsome cloth, 10s. 6d.
A DICTIONARY OF DOMESTIC MEDICINE AND HOUSEHOLD SURGERY.
By SPENCER THOMSON, M.D., L.R.C.S. (Edln.), and J. CHARLES STEELE, M.D., late of Guy's Hospital.
'Thoroughly Revised and brought down to the Present State of Medical Science by ALBERT WE ST LAN I). M ? A ?, M.D., and GEO. REID, M/D., D.P.H*
With a Section on the Maintenance of Health and the Management of Disease in Warm Climates by JAS. CANTLE, M.A., M.B., F.R.C.S.; and an
Appendix on the Management of the Sick-room, and Hints for the Diet and Comfort of Invalids.
From the Author's Prefatory Address.
" Without entering upon that difficult ground which correct professional knowledge and educated judgment can alone DPrm't to be safelv trodden, there
-is a wide and extensive field for exertion, and for usefulness, open to the unprofessional, in the kindly offices of a true DOMESTIC MEDICINE, the timelv
lielp and solace of a simple HOUSEHOLD SURGERY, or, better still, in the watchful care more generally known as 'SANITARY PRECAUTION,' which
tends rather to preserve health than to cure disease, ' The touch of a gentle hand' will not be less gentle because guided by knowledge, nor will the sa,te
domestic remedies be less anxiously or carefully administered. Life may be saved, suffering may alwavs be alleviated. Even to the resident in the midst of
civilisation the 'KNOWLEDGE IS POWEIl' to do good ; to the settler and emigrant it is INVALUABLE."
"The amountJof Useful Knowledge conveyed in this Work is Surprising."?Medical Times and Gazette.
LONDON: CHARLES GRIFFIN & CO., Ltd., EXETER STREET, STRAND.
iv THE HOSPITAL. Oct. 3, 1903.
Publications?continued.
JOHN WRIGHT & CO., ? BRISTOL.
Full Cata 1 ogu^ of MetHcal^l^blk n Application.
Nearly ready. Demy 8vo 9s. 6d. net.
ESSENTIALS OF PELVIC DIAGNOSIS WITH ILLUSTRATIVE CASES.
Bv E. STANMORE BISHOP. F.R O.S.Hng . Author of "Uterine Fibromyomata, their Pathology, Diagnosis, and Treatmeat." Hon. Surer. Anciats
Hospital, Manchester ; Vicfi-Pre?ident British Gynaecological Society Londso ; Ex-Pre*ident C inical Society, M*noti33tir. eic. And an APPENDIX
OK EX4.MINA.TION OP BLOOD, ETC., by CHA.S. H. MELL1ND, M.D.Lond.,, M.R.C.P., Hon. Physician Aucaata Hospital, Manchejter; Pla:t
Physiological Eclialar, etc., etc.
Just published Fifth Edition Reviled and enlarged. Manv Illustrations. 7s. 6d. net.
LECTURES ON MASSAGE AND ELECTRICITY IN THE TREATMENT OF
DISEASE. Revised. Partly Rewritten and with much additional matter. ByTH03 STRETCH D~)WS3 M.D Abd. FR.C.P.Ei.
Just published. Third Ttdition, Revised and. enlarged. Flexible leather, gilt, 6s. 6d. net; or interleaved for notes, 8s. 6d. net.
THE POCKET THERAPIST. A Dictionary of Disease and its Treatment: being a concise
manual of Modern Treatment, and an Aid to Memory for Students and Practitioners. By T. STRETCH DO'VSE, M D.Abd., P.R.O.P.Ed.
Just published. Demy 8vo. 2s. 6d. net.
OBESITY. The Indications for Reduction Cures. By Prof. Dr. CARL YON NOORDEN, Physiciaa-
in-Chief to the City Hospital, Frankfort-on-Main.
Just published Demu 8vn 2s. 6d .net.
MEMBRANOUS CATARRH OF THE INTESTINES. (Colica Mucosa.) By Prof. Dr.
OARL YON NOORDEN, Phjsician-in-Ohief to the Oity Hospital, Frankfort on-Main.
Just published Demu 8i'o. 3s. net.
NEPHRITIS. By Prof. Dr. CARL YON NOORDEN, Physician-in-Chief to the City Hospital,
Frankfort on-Main.
Third Edition, Twelfth Thousand. Small 8vo Illustrated usith ?00 Original Drawings. 2s. 6d.
LANTERN RLTnjtS OF THE ILLUSTRIOUS, Is. and Is. 6d. each.
" FIRST AID " TO THE INJURED AND SICK : An Advanced Ambulance Handbook. By
F. J. WARWICK, B.A.. M.B.; Surg.-Oapt. Volunt. Med. Staff Corps and Armv Med. Reserve of Officers; and A. C. TCNSTALL, M.D., F.R.C.3.,
Surg.Oapt. East Lond. Vo'unt. Brig. Bearer Co ; Lect. and Exam, to the the St. John Ambul. Aeso).
" The Authors are both Volunteer Med. Staff Officers and know their subject. The book is simply written "?Lancet
'? An excellent handbook treats the subject more seriously and fully than the majarity.. ..To all teachera most usjfal, and to student3 may well
serve as an advanced text-book."?Brit. Med. Journ.
Third Edition, now Ready. 2s. per Sheet, or 27s. 6d. net. the set of 16 on roller.
(ADOPTED BY THE WAR OFFICE, THE ADMIRALTY, AND THE LONDON SCHOOL BOARD.)
LARGE " FIRST AID " DIAGRAMS: Being Enlargements of the Principal Illustrations in
the above book for Lecturing Purposes and Class Illustration. On sheets 26 in. by 40 in.
Second and Revised Edition, Large 8vo. With 8 Full-page Plates and ovr 5'0 Original Illustrations drawn by the Author, and numerous Woodcuts.
680 pp., 10s. 6d. *et.
OPERATIVE AND PRACTICAL SURGERY: For Students and Practitioners. By
THOS. CARWARDINE, M.S.Lond., F.RC.S., Assist. Surg. Bristol Rov. 'nficm. With Special Chapters on the EYE, EAR, and TEETH, bv
M'ssr*. F. R. CROSS, W. H. HARSA.NT, and W.R. ACKLAND ; on the NOSE and LARYNX, by Dr. P. WATSON WXL.LlA.Md ; and SURGICAL,
BACTERIOLOGY, by Dr. J. O. SYMES.
Demy 8to. Cloth Round. 3s. 6d. n't.
MEDICAL ETHICS: A Guide to Professional Conduct for Students and Practitioners. By ROBERT
SATJNDBY, M.D.(Edin.), F.R.C.P,(Lond.), LL D.
Fifth Edition Reprint. Revised and. par-ly Rewritten, with Additional Chapters, and264 Illustrations 10s. 6d.
PYE'S SURGICAL HANDICRAFT: A Manual of Surgical Manipulations, Minor Surgery,
&c. With Special Chapters on AURAL SURGERY and the TREATMENT OF TEETH, by Messrs. FIELD and SIDNEY SPOKES. Revised,
and Edited by BERTRAM M. H. ROGEKS. B.A., M.D., B.Oli.Oxon.
Waistcoat Pocket Size. Cloth Covers, Is. each, iiost free.
"GOLDEN RULES" SERIES
I IN MEDICINE AND SURGERY. .
1. Golden Rules of Surgical Praetice. By
E. Husky Fen wick, F.R.O.S. Eighth Edition, revised anc^eil ir^ed.
2. Golden Rules of Gynecology. By S.
Jebvois Aarons, M.D. Fourth Edition.
3. Golden Rules of Obstetric Practice. By
W. E. Fothhrgill, M.A., B.Sc., M.D. Fourth Edition.
4. Golden Rules of Medical Practice. By
A. H. Evans, M.D., B.S., F.R.O.S. Fifth Edition.
5. Golden Rules of Psychiatry. By James
Ehaw, M.D. Second Edition.
6. Golden Rules of Physiology. By I.
Walker Hall, M1B., Oh.B. (Vict.), and J. Ackworth Menzies,
M.D. O.M. (Ed.). Secotd Edition.
7. Golden Rules of Ophthalmic Practice.
By Gustavds Hartriege, F.B.OS. Second Edition.
8. Golden Rules of Skin Practice. By David
Walsh, M.D.Edin. Second Edition.
9. Golden Rules of Aural and Nasal Practice.
By P. B. W. De Santi, F.R.O.S.
10. Golden Rules of Hygiene. By F. J. Waldo,
M.A., M.D. (Oantab.), D.P.H.
11. Golden Rules of Children's Diseases. By
George Oarpektfr, M.D., M.R.O.P. Enlarged Double Number, 2s.
12. Golden Rules of Refraction. By Ernest
E. Madeox, M.D., F.R.O.S.Ed.
Eighth Edition, Revised, 8vo. thiek paper covers, 1/6
op cloth, 2/6.
**A.Y BE SAFFJ-Y RECOMMENDED.
OUR BABY: A Z"Trs
By Mrs. irANGTON Hewer, late Hospital Sister.
"We can heartly recon>meixi it to those who are responsible for the
health'and hippiae' s of infants.Iirit. Jled. Journ.
??The base we have evtr seen."?Edin.,Med. Journ.
Nearly Four Hundred Thousand have been Issuein
Samples and?Prices Free.
WRIGHT'S CHARTS & CASE PAPERS
Are. among the Best and Cheapest published.
Hospital' Charts?Clinical Figures?Pocket Charts?4-Hour
Charts-3-Week Charts - Large Scale Charts?Nursing:
and Diet Charts, Case Papers, etc.
1,000, 22/-; 500,12/6 ; 250, 7/-; 100, 3/-; 50, 2/<- ;
and upward?.
Samples and Prices Free.
IMPROVED CHART HOLDERS.
With Nickel-Plated Fittings. Cheap, Handsome and
Durable.
In Leatherette from 1/-, or Celluloid from 2/- each.
MEDICAL ACCOUNI BOOKS. Samples and Prices on
Application.
PRESCRIPTION BOOKS. 1/- each ; 10/- dozen.
CERTIFICATE BOOKS : For Use in the Case of Clubsr
Scholars, &c 100 leaves, 1/6 ; 250 leaves 2/6 each.
DISPENSING LABELS. Lithographed, 2/-'per 1000;
Letterpress, 1/6 per 1,000.
Bristol: J. WRIGHT & CO. London: SIMPKIN & CO., Limited.
Oct. 3, 1903. THE HOSPITAL.
Publications?continued.
CASSELL & COMPANY'S ANNOUNCEMENTS.
ENTIRELY NEW AND REVISED EDITION.
A MANUAL OF OPERATIVE SURGERY.
By SIR FREDERICK TREVES, Bart., K.C.V.O., C.B., LL.D? F.R.C.S., &C? &C.
Revised by the Author and JONATHAN HUTCHINSON. TUn? F.R.O.S., Surgeon to the London Hospital; Examiner in Surgery, Royal Army Medici I
Department. With abiut 450 Illustration*. Vol I. Noic Ready. Vol II. Iieady Shortly.
Complete in Tito Vols., 42s. Supplied in sets only.
OTHER WORKS BY SIR FREDERICK TREVES.
Surgical Appl'ed Anatomy. Revised with the Assistance of ARTHUR KEIIH, M.D., F.R.O.S. With 80 Engravings. New & Enlarged Edition. 9s.
Intestinal Ob Vruction. With 118 Illustrations. 21s.
The Student's Handbook of Surgical Operations. With 94 Illustrations. 7s. 6d.
READY SHORTLY.
ELEMENTS OF SURGICAL DIAGNOSIS. A Manual for the Wards. By A. PEARCE GOULD,
M.S., M.B., F.R.O.S. New and Enlarged Edition. 9s.
NEW A.ND REVISKn EDITION. NOW REiDT. Prio9lOs.6d.net.
TROPICAL DISEASES. A Manual of Diseases of Warm Climates. By Sir PATRICK MANSON,
K.O.M.G., M.D., LL.D.Aberd., F.R.S , &o? &c. With Two Coloured Plates and 130 Illustrations.
NEW ANr> REVISED EDITION. JUST PUBLISHED. Price 21s
TUMOURS, INNOCENT AND MALIGNANT: their Clinical Character and Appropriate Treat-
ment. By J, BLANDSUTION, F.R.O.8., Surgeon to the Chelsea Hospital for Woman ; Assistant Surgeon to the M. id diet* x H?ep tal, Lonlon.
With 312 Illustrations.
NEW AND REVISED EDTTIDN. NOW READY. Pries 10s. 6d. net.
DISEASES OF THE SKIN. An Outline of the Principles and Practice of Dermatology. By
MALCOLM MORRIS, Con suiting Surgeon to the Skin Depaitment, St. Mary's Hospital, London, With Two Coloured Plates, 36 Plain Piatf s and
a number of other Illustrations.
DISEASES OF WOMEN: a Clinical Guide to their Diagnosis and Treatment. By G. ERNEST
HERMAN, M B Lond., F.RO.P With upwards of 260 Illustration*. Ne^v and KsvUpd E litio i. 25s.
A MANUAL OF MEDICAL TREATMENT, OR CLINICAL THERAPEUTICS. By I. BURNEY YEO,.
M.D.. F.RO.P. New and Revised Edition. With numerous Illustrations. Two Vols 21s.net.
FOOD IN HEALTH AND DISEASE. By I. BURNEY YEO, M.D., to. 10a. 6d.
SURGICAL DISEASES OF THE KIDNEY AND URETER, INCLUDING INJURIES, MALFORMA-
TIONS, AND MISPLACEMENTS. By HENRY MORRIS, M.A., M.B.Lond., F.R.O.S. With 2 Coloured Plite3, and upwardi of 203 IllQittd-
tions. Two Vols. 42s. net.
MATERIA MEDICA AND THERAPEUTICS: an Introduction to the Rational Treatment of
Disease. By J. MITCHELL BRUCE, M.A., LL.D., M.D., F.R.O P. 7s. 61.
A Complete List of MEDICAL WORKS published by Messrs. Cassell <$? Company will be sent post free on application.
CASSELL & COMPANY, LTD., LA BELLE SAUVAGE. LONDON, PARIS. NEW YORK, AND MELBOURNE.
THE BRITISH JOURNAL
OF
CHILDREN'S DISEASES
Edited by GEORGE CARPENTER, M.D.
THE First Number of this Illustrated Monthly Journal, the only British Journal specially devoted to the
subject of Children's Diseases, will be it-sued in DECEMBER.
The British Journal of Children's Diseases will be printed in large and clear type, upon good
paper. Twelve numbers will form a handsome Royal octavo volume.
SUBSCRIPTIONS, 12s. per annum post free, are now being raceived by the Publishers,
ADLARD & SON, Bartholomew Close, London, E.C.
JUST ISSUED. EIGHTH EDITION. Crown 8vo, 10s. 6d.
PHARMACY, MATERIA MEDICA
AND THERAPEUTICS
ii now rewritten and printed in large type, and is made a oompanlon to
the following volume. It contains an account of the action of every
drug In the B.P., as well as a section upon all the newest drugs, with
an Introduction to the Art of Dispensing. With woodcuts, prescriptions, & a.
Also FOURTH EDITION. 1055 page*, 8vo. 16s.
DICTIONARY OF TREATMENT.
Containing a Revised and up-to-date Article upon every Diseased Con-
dition, whether Medical, Surgical, Gynaecological, or Ophthalmological,
as well as Articles upon Poisoning, Drowning, and the various Surgical
Emergencies and Accidents, arranged for Rapid Reference.
By W. WHITLA., M.A., M.D., Professor of Materia Medio*, Qaeen'i
College, Belfast; Senior Physician to the Royal Victoria Hospital, &o.
The Publisher will Bupply both volumes, carriage paid, for 21/-
London: HENRY RENSHAW, 356 Strand.
THE HANDBOOK OF THE OPEN-AIR TREATMENT
By Dr. Charles Reishardt
(Visiting Physician to Hailey Sanatorium, Walling ford, and Stourfleld Park
Sanatorium, Bournemouth),
Contains Views, Descriptions, and Information respecting
30 Sanatoria in Great Britain.
Price: Cloth, 2/6; Paper, 1/- (postage 4d.)
The " HEALTH RESORT " Publishing Office, 379 Strand, London, "W.O;
WORKS BY DAYID WALSH, M.D.
RONTCEN RAYS IN MEDIOAL WORK. Third Edition. Ill, M,
BailliSre & Cos, London.
THE HAIR AND ITS DISEASES. Z8.6d.net. Baillifcre 4 Cox.
GOLDEN RULES OF SKIN PRAOTIOE. ls.net. Wright <h Co., BrirtoL
ACE AND OLD ACE: a Handbook. 2e. 6d. R. A.. Everett & Oc., LocdoB.
vi THE HOSPITAL. Oct. 3, 1903.
P U b I i cat IO n S?continued.
JUST OUT. Price 5/- net.
CANCER:
Its Causation and Treatment without
Operation.
BY
ROBERT BELL, M.D., F.F.P.S.Glasg.
Consulting Surgeon to the Glasgow Hospital for
Women.
London: BAILLIERE, TINDALL & COX,
8 Henrietta Street, Covent Garden.
Now ready, SECOND Edition, with numerous Illustrations
and additional Chapters, crown 8vo., 5s. net.
Mentally-Deficient Children;
their Treatment and Training,
By G. E. SHUTTLEWORTH, B.A., M.D., &c.,
recently Medical Examiner Defective Children, School
Board for London; late Medical Superintendent, Roya,
Albert Asylum for Idiots and Imbeciles of the Northern
Counties, Lancaster; formerly Assistant Medical Officer
Earlswood Asylum.
" Altogether there ia no book on the subject so compact and so com-
prehensive, in which the busy practitioner will find all he might care to
know of the subject in a condensed but scientific form. The book is well
illustrated."?Edinburgh Medical Journal.
London: H. K. LEWIS, 13G Gower Street, W.C.
JUST PUBLISHED.
An Important Pamphlet on
THE MEDICAL ATTENDANCE OF LONDONERS
An Economical System of Medical Relief freed from existing abuses and adequate to protect all classes and interests.
\ By SIR HENRY BURDETT, K.C.B.
" Price 1 /-
Obtainable of all Booksellers, or of
THE SCIENTIFIC PRE3S, LIMITED, 28 & 29 Southampton Street, Strand, London, W.C.
Supplement to " The Hospital," April 2, 1904.
A JOURNAL OF
THE MEDICAL SCIENCES AND HOSPITAL ADMINISTRATION,
INCLUDING
A SPECIAL SECTION FOR NURSES.
IN TWO VOLUMES ANNUALLY.
EDITED BY
SIR HENRY BURDETT, K.C.B.
VOLUME XXXV.:
OCTOBER 1903 TO MARCH 1904.
Xonfcon:
PUBLISHED BY THE SCIENTIFIC IPRESS (LIMITED), AT THE HOSPITAL [BUILDING, j)
28 & 29 SOUTHAMPTON STREET, STRAND, W.C.
JJ
. Supplement to " The Hospital," April 2, 1901.
ii Index to VoL XXXV. THE HOSPITAL. 3rd October, 1903, to
INDEX TO VOL XXXV., 1904.
*,? Articles under the general heading Hospital Clinics and Medical Progress are distinguished by the initial CO.) for Hospital Clinics, and by (P.) for the
rest of the series. CW.) distinguishes the Institutional Workshop articles, and (A.) the Annotations.
Abdominal Pain 'PA 152
Abdrm'nal Sn'fe'V Prosress in (P \ "59
Abiofita Hepatic, Treatment of ("0 ) 405
Add'so^'a Tlnease' P crmentation in (A..), 3C9;
Treatment of 0 ) 30
Alcoho' a*"d Fvolntiori (\ ), 269
Alexandra hospital 'or Hhi'dre", Phyl (W.), 2R0
Alonecia Areata ( A ), 441; Etiology of (0 ), 446
Ambidf-x^e-ity. 4^8
Amb d- xtral HnHure Society ( A ) 337
Ambidextral Work. Physiology of Simultaneous
(C.) 454
Ambulances for London 399 (W) 413
Amuremen*<> by D^atb B'sVs. ( \.) 359
Ana;mla ; Pernicious (P.). 392 : Snlen!c (P ), 392
Acse-thetics. Proffps" i" (? ) l'O
Anastomosis. Tr-testin^l (P.), 29?
Aneur' sm; Abdomi al p.). 276; of the Abdo-
minis Aorta (C.) 224; Gelatin Treatment of
(P , 3?3
Ancina Pectoris CP ?> 2P0
Anima1 Parasites ""P.). 188
AnVylostooiiasis In Or-?"all 42
Aorta, P"lsative (P. 276
Apolipv for Cheapness, *n C t.), 221
Append'c'tis; Mortality o' (H.). 291; Treatment
of (a .187; Value of Blood Examination in
(D.), 30
Araund th- Ho-pit*ls (W\ 73
Arsenic in Oream of Tartar ' v), 459
Arsen cal Commission. Th?, 18'
Arterial Hyp?rteasion P ) '57
Arterial Sclerosis and veryous Discus' 'P.). 133
ArthriMs: ^norThoeM. and Ophthalmia Neona-
to-um (0.). 6S ; PheumatoH T. , 408
As irator. A n'w form of P.) 118
Asthma (P.) 448
Asylum. Deaths in an (A.), 401
Atlas. Fracture of the. and Odontoid Process (P.),
448
Atrophy, Muscular, an*! Sclerodermia (0.), 445
^ab'es and B?auty Doctors (A) 329
Hicllli Tnh?r>le. Passage of, through Intestinal
Wall (P > 373
Bao'l ui. Tie Fusiform (P.\ 85
Bact?r'ology Prog-ess in (P.). 85, 151 189, 372.
408_
Bilbarziofis "f the Ge ito "rinary Organs TP \ 82
Birth-rate nd Pea*h--at? in ^rg^ntini (A.), 163
Bir'hs, Marri?g?s, and Deaths (A.), 145
Bladder, Tumours' f th O.), 80
BMnd Amusements for tho , 309
Blood, Bic'pr'ol-gy of the (P. . 152; Progress in
Diseases o* th? P.) 392.409
Blo^d Pros=ur=>; Reaction (P.), 49 ; T>ice 0f, jn Later
Life fP ), P45 ; Vacations in P.), 260
Book World?
Advpr'iserj A.B O.. 339
B'sbop Ess n'ia's of P?lyle Diag-osis, 120
Burrell, Co nc'lman, ard Withi?gton. Medical
and Pure ca> PDO't3 of the Boston Oity
Hospital, 1903. 279
Oaril'o. Cancer 296
Carpenter. Bri'ish Journal of Children's D's-
e sea. 262
Carpenter GiHen Rule3 fir the Diseases of
Children 136
Ohatta w. D g*st of Researohps. bearing on
the Briti'b Pha'mac 'nae'a. 1898. 230
Col'fe ?nd Wierhtm n. First *id 154
Councilman. See Burrell
Crandall. H w Kmp We". 450
Ciillineworth. Ohar'es "Vhita. MD, 450
Cunninsr. AidR to 0u*g'rv. 450
Doctor's Diary 1901 231
Dotsp. Lfcture3 on Vlassjga and Electricity,
135
Dunlop. Dr. John Brmyn 293
Duprat. Mora's a """reat se on the Psycho
Soci "logical Ba"es of E hiss, 375
E^sar. P<-aotica of bstetrt^s 230
Fvm r. Scheme of B-ain ^toroga, 358
Furneaux *e1i'ed by PMlip'3 Anatomical
Model of the Female Body, '6
Gadd. Drugs, tbeir Production. Preparation,
and Properties 375
Gould. Elements of ^u-gioal Diaenoai3, 210
Oraha-n. rr? ?tis? on M iss ? ere, 318
Guy's Hospital 1724-1902, 339
Hall. Tho Medical Examination for Life A.s?ur
a/ice. 133
H*'l Purin Bodies of Food-Stuff*. and R6ie of
^ric A.cid i" health aid Disease 333
H>r-ing, Sta-ilisatioi of Urethral Instruments.
351
H-scheJl. Manual of Intragastr'c Technique
135
T'lHer. Prevntioo of Cinsunontion, 3"8
International Oli <'c3. Vol. TT . 36
Jeliett ?hort Practice of Grvnseiolngv. 7 39
TC?lly International Clinic*. Vo! II.. 279
Kocker, Te^t-bi k of Ooerativn SurJrpr7. 154
Lane. Frj's Royal 'Ju;de to London Charities,
4C0
Macdonald. Th= Re'igious Sense in its ^cisntifio
^BflPCt. 291
Mol'and. Pocket Book nf Clinical Methols, 279
Ma>r>niorp Afte" Treatment of Operations 210
Murnhv Tubnrculn-is of the Female Genitalia
and Periton *u-n. ?19
Noorden Clinical Treat's v, 120
Over?nd. Animal Ph*sioinev Di?P""am3,154
Poo s Roflaxs on Bural Hygiene, 36
Practical Adverti^inc. 154
Prendervi'ie. E'hvlohioride in Surgical ani
Dental PracHen 279
Preston See Wright
Riviera an" Ttalv fo" a ?10 note. 318
Roererp. Loca' <3ov rnm^nt Annual and Offlsial
Directory. 450
Shaw. Ph' siognoonv of M>ntai Diseases. 262
street's "pwsoapar Direitfv. 376
I1 ipD. B^an'i'ul Biakra 311
Vansrhan. Principles ani Practice of Surgery,
262
Vincent Nutrition of the Child 311
Walter. The Wxant, "Vence of Health based
upon Life's Or?at. Law, 293
Who's Who, 19^4 450
Who s ^ho Year B"ok 450
Willing'" nresa Grti'd?. 1904. 318
Withington. See Burrell.
Wright and Preston, Handbook of Surgicil
Anat"mv, 210
Year Book of Scientific and Learned Societies.
190*, 230
Brain an" Verves, Progress in Surgery of (P.\
316. 336
Brain Tumours, ?urgica' Tr?atmenv. of (P.) 117
Bripht's isease, Surgical Trsatm-nt of Chronic
(P \ 315
British Tn?t''tn'i"n? for tV Core of the Inebriate
'Wi 37 7'.137 159,248 263
Bronchial Tubas, ' 'ila'at'on of the, in Children
CO. , 130
Pronchit's Fbriwva fP >. 119
Burial, ''remat-ure C V 1 3P5
Burma. The Hospitals and Dispensaries of (W),
249
Calculus. Tmoacted and some oth?* CiU3'S of
Obstructs > in the TTi-etors (P.). 335
Cambridge VevSo'e ce Bnildings at, (W.) 410
C'nal culi, L'crati >n of ttie P .
Hancor; ?rigi' of 200; Progress in fP.) 2"!6,
221: of th? Prostate P. . 82, (0.), 115 ; of ths
Sto-nacb (P ), 16
Cancer Research ; 3 '7; Progress of. 5
"arbolic *c>d and the Treatment of the Plague
(C. . 46
Carbo'ic Acid, "^h" Do=e of t v.). 77
arcinoma of the Breast, Operations in (0.), 46
Da'cin ima, On the ?1 i logv of (0.), 313
"'ardiac \.neurv?ms CO 45
0 rdiac Mnsc'e (0 >. 389
narol of ?ha Maiso i Dini, 278
Carehellir \ta*U, Congeoit il P.), 100
Cerebral Soinal and -ferve Su-gery, Progress in,
rp.) 441. 465
Cerebral Turn'ur, Two Cues if. Successfully re-
moved by Oifra'ion 'P.") 316
Cerebr i Soinal F aid in (Je '?ra! Paralvs's 0.\ 14
Hervica' Spine, Fracture Uislic *tlon of the, with
oat Para'ysis P ), 50
Charing Cro's 'o* >i a1 Imp' vements at (W.), 39
Hh tritv and Wisdo n f A ) 2 V7
Coild'en. Dio-ase' f P.) 99
0aildr?n'* nnum ttee of the Metropolitan Asylums
Board, 155
OhTlren's DNeasea, Ptudy of( K.). 253
Chloroform The Administration of (0 ), 186
Oho'ec at'ctomv P ) 67
OholelithUsia fP ) 51
Ohorea, Treatment of 'P.) 461
HircuHting Libraries for Poor-law Patients (1.),
163
Cirrhosis, Operations for the Aaciti' of CP.^ 37^
OiviHsaMin?Ta Oar Civilisation a Failura? 297
Oiaes'pa aa Schoo' Booka ( V). 287
Hef* Palate. wh?n t ? Operate in OA 13
Ood liver Oil, The Revolt? of (A. . 183
Col tia. The ''urgical Treatment of 'O , 13
Colon. Perforation of the, bv Smilj Foreign Bodies
fO \ 99
Constipation, Non-med'cinal Treatment of (0.),
170
Oona^rnoMon Notes (W \141 174. 2*0
Consumption ; Eirly Diagnosi1 if ,0.). 371; Sana*
t r'um Trfatm?nt ^f 0.), 387
0,naumptiva ; The Education o? the, 126
Consumptive3, Treatment and car^ of. at their
ffnmea fP \ 102
Oonvul?iona 'P ) 100
Oonper. TTse of. in Syphil's (0 \ 99
Cornea, no"io<l. Onerati"na for (P.).246
Ooroner's ,Tn y The (K.) 201
0 ^gt of Smali-pnx The, 1
O'eraat'on CP >. 314
n immal Reapinaibiiity of the Tnaa^e, 25'.
Cyanosis. Ohronic, with Polycythsemi i (P.). 392
dyst'tis. Pyeilti . and Pyelonephritis (P.). 151
O.TSfciRcoiy (P.\315
Cystoscopy and Cryo3copy (P.), 293
Danish Mdical Institutions fW.), 22 57,13?, 264
Deafness and Rlind-ess (P.\ 447
nPnga? F'ver CP.) 237
DeTnaMogy P'Ogresa in CP.">, 14, 4,.4. 443
Dermoid", Ovarian and Pelvic 0.). 370
r>pt'riorat!on of the Breed, 104
Diabetes, Treatment of (0 ), 63 Prog'e33 in, 227,
244
Digest!?p Orean', Progress in Disaisei of (P.), 132,
15? 169 188
Diphtheria (P.', 407: Import ce of Recognising
Mi'd Forma of. 252; Relation of Mild Types
of to Publ'c Hea'th (0.\ 22o
Dirty M'ine^ and Diaease ( ^ ) 77
Disinfecion Difficulties at Liveroool ( O, 43
Dislocation of thejOuter End of the Clavicle (0.),
98
" Doctor," Th?, 249
Doctors and Drug Supnly, 347
Doc o s and Hid fives (A..), 267
Dr ?wning P .103
D'uea ?nH War ( a.). 385
^uat-Wecting in Oprmany (A..), 319
Djeentery (P.), 169, 207
Ectopic Citation (P.">, 354
Editor's L>t?r Box. 91 103 123, 141,160, 234, 305,
3% 36? 382. 397, 437
"Elect ioal" Quacks ( V.), 61
Rlecrlcitv ani T'gh-FrJqnencv Currents CP.), 48
Electro-'heraDau'ics Tlie ""uture of (A.), 269
Emotional Donor, The < A,), 3
"ndoca'd tia Infective CO. . 1?9, 147, CP.), 260
Enuresis: 1n Children (C.) 334 : Diet in Nocturnal
(\ >. 367
Epi'epiy(P.) 191; and Crime 199
Hpistwia P \ 425
^thvl Ohloride as a General Anaj'Hietlc CO V 148
Exp rime >ts ni Vnim is O 165 i85, 203, 223,
242 255 271, 289, 311, 331. 351
Wye, Bicterioiogy of the CP.). 151
Eyeball, Dislooa-ion of tha (P.), 66
Facial Paral ais : Following E notion (A.), 145 ;
Surgical Treatment of, by Nerve Anastomosis
CP.), 336
"ashion in Foods CA.), 221
Feeling: and Disease, 93; The Science and Art
of. 95
Fever : Kntaric, Tnfectivity of. 383; Rheumatic,
Acnfe P.l, 372; Yellow (PA 391
Fevers, Progress in (.P.), 207, 274, 294, 374, 390,406
Supplement to " The Hospital," April 2, 1904
26th March, 1904. THE HOSPITAL. Index to Vol. XXXV. iii
First Garden Olty. The fW \ 73
Tood Adulteration in 1902,143
Fool : Choking and Catering, 108
Pood of the People, The, 110
Food* and Taxation, 60
Freedom of the Profession, The, 28S
Oal'-Bladder Operations on the 'P.), 374
Hal -Stones, Patholozv of CO.), 31
Gangrene of the Le? following Pneumonia '0.\ 64
Gaseerian Ganglion. Kxcisioi of, for Trigsminai
Neuralgia (P.\ 465
Gastric: O'ises P.), 152, 191; Teta-iy (P.), 132:
UIc?r (P ), 132
Gastro-Rnt irostomy (P.), 32
Geni^o TTrlnar? Surgerv. Proeress In (P.), 82, 293
German Emperor. The, (V). Ill
Glance" at the Hospitals (W.), 23,39,57, 217,265.
285 324, 351
G'anrts, Lynohatic, in Cheek (0 ) 41o
Goiococcus Infectioa; Di?Eus? (C.) 81; In Infant
(0.) 167
Gonorrhoea in ???he Female (P.) 754
Ouild of " The Poor Tilings " ( V.), 319
Gyneecjlogy, Progr;s3 in (P.),338, 334
""achiah in thi Old Testament. 52
Hasmatirrhich's. T'aauatii (0 ), 3??
?"le-n'-erlobmuHi. Paroxysmal (U.), 64
Headaches (P.) 427
Health of London. The, 235
Health Preservation among Weimar Schola's (k.\
163
Health atat'ons, British (W), 361. 379
Heart and Circulation, Diseases of tie, (P.), 223,
245. 26 \ 275
Heart TiSolee; Boldness in Treatment of (0.),
370; Prevention of, in Child-<>n (CI."), H
"Heart D'seas^s Progress in 'P.), 356 393
Hemiplegia. Progressive Developing (P.), 133
Hepactomy, Pa'tial (P ) 373
Hepatic Oi'rhosis (P ), 67
Hernia ; Unusual Oa^e of (O.), 243 : Ventral, Pre-
vention "f, after Laparotomy (P.), 253
Hiccough (P ), 132
Homeless Poor, 457
Hospital fo" Infec'ioas Dis^a'e*, Sheffield (W V 341
u Hospital," The Library and Charities Bureau ( V.),
198
Hospital Meatings (W ). 140, 315, 393 415, 431, 454,
463
Hosoital and G n?ral Pr ctiMone"*. 366
TTospita's Association, The fW.), 394
Hour-g'as ctomach (P.), 295
Housing of the Working Classes (P \ 134,150
How to get rid of the Tramp V \ 27
Hygiene and Temperance, Teaching of, to School
Children, 328
Hyperchlorhydria (P.1,132
Hypnotics. Dancers of (P.\ 133
Hypochondriasis, Ytultiole and Alt.matin; Per-
sonalities in (P.), 101
Idiosyncrasy in Re'ation to Dovs (O ), 205
'Inebriate, Fliminat'on of the. 94
Infant; Degenerate (0.1, 369 : Weeding (P ) 99:
Feeding by Home-made Humanised Milk (O.).
2o6
Infection, Continuous L-'cal CO.), ?56
Inflat'ng the Stomach, Tne Dangers of (n.\ 273
Insane, The Pto'y of the, from Year to Year, 304,
346 365.379
Insanity, Tin alleged Increase of (A.\ 3
Inspectors Lady health in Kensington. 400
Tnsuranca aeainst Surgical Operations 440
Intestinal Obstruction <P. . 295
Tntestines. Progress in Surgery of the (P.), 2P2
Intra-cranial ibscess and Middle-ear Disease (O.),
65
Intravascular A.ntis^psis (P.\ 8o
Intussusception (P.), 101
Kaiser, The (A ), 127
Ketline Sanatorium, Holt, Norfolk, The (W.), 107,
123
"Kidney Movable (O.), 257 ; Horse3hoe, Operation
for (P ). 335
Kiineys Inj <r fs of the (P.), 316
King Hdward VIC Sanatorium. The (W.\ 107
King Rdwards Ho-pital Fund for London (W),
211. 428
King's College Hospital; 41, (W\ 53; Removal of
lW.). 451
Lantern Test for Oolour-visinn (PA 259
iLeague of Mercv. THe (W ), 231, 430, 456
Leishman's Bodies (P.), 403
iLsrrosf PA 446
LeuUtctnia P.). 409
Pliability of Voluntary fchools for Negligence,
The, 236
Light Onre.''Bed Crcse,"atd Electrical Institute
(W.J, 433
Liver and Gall bladder : Diseases of the 'P.), 183!
Progress in Surgery of the (P.\ 50 67
London HospPals, The (W.\ 24? ; historical
Sketch's of some of CW.'? 343 377 : with
Medical Pohoo's and their ?ite? (W , 193
tontfon School Board and Public Health, The, 192
London's Ambulance Association (W.), 334
Looking B\ok, ?35
Lymph in tie Tissues (P.), 409
Macnglo'sia (0 \ 143
Malaria CP.). 189 294
Massage by the Blind (A..), 181
Mastication (P.), 132
Materia Medica aad tin Mudicil Curriculum, 7o
Maternal ResponsibUitv (A..), 43
Medical A-ppointments, 259
Medical na?e of Children In Elementary Sshoals,
The. 220
Medical Council and the Royal College3, The (A..),
183
Medical Education an I the University of London,
418
Medical Teachinr in the fifteenth Centu-y, 23
Medicine and Law ( A.). 4 9
M'dici-Legal qocie* v, The < A..), 237
M'Jrena Neonatorum P.)i 101
Meningitis. Past Bisal due to Pnaumoaocsus
Lanceolatus <C.), 463
Metric Svsteu In M dicina. Th?. 343
Metropolitan Hasoital Sunday Funl (W.)> 215
Micro-orgaiisaas in the A.ir (P.), 190
Midlives ? ct The. 417
Milk, Municipal, Supply, 327
Modern ?ro3nival and Institutional Fittings 'W.),
72.105,173
Hodsrn Sociology 70. 104,15^ 297
Moral Education of C lildren ( v.), 237
Mortilitv of Infectious Diseases. Tm, 161
Mount Ye^non Hospital for Consumption, The
( v.) 90
Muca-Memb-aneous Oalitis (P >, 153
MultiD'e Consciousness, Extraordinary Form3 of
P.), 116
Murmurs, Diastolic Basis, withauH T>)loas of
Aortic or Pulmona-y Valves (P.), 333
Muscle Tonus and Tend ia "heuomsna (P.), 133
Musaular Movements (P.), 208
National Defence, The Nob'e Art of ( V,), 3C9
Nauheicn Tre it nent, The (P.V 357
Nee'les, Rx'-.'-action of, after Lacjtion with X rays
(0.), 463
Nephritis ; Chronic TntersMtial (P.) 465 ; Oh-onic\
without, Albuminuria (0.), 29; Treatment of,
(0 ). 148
N*rve Cells, The Histology nf (P.) 467
Nerve Surgery, Progress in (P ) 34, 49
Nerve Suture and Nsrve Regeneration (P ) 3*6
Nerves; Injuries of the (0.). 167; Saoi-teiing
l?rge, in Amputations, the Necessity for (P.).
49
N8rvon3 System, Progress in Diseases of the (P.),
463
Neurology, Progress in (P.). 117.133. 208, 423
veuron?s, Toxic Deganeration of (P.), 134
Naw App'iances and Things Medica', 19,35. 51, 69,
8\ 103. 119, 135. 153 209. 223, 246 251, 277,
295, 317, 333. 357, 427, 449
New Complexions for Old (A ), 61
1903 ( i) 239
Notes and ew, 4, 58, 44, 62, 78, 93, 112, 198, 146
164, 184, 202, 222, 238, 254, 270, 288, 310, 33'J,
350 3S8, 386, 40?, 420, 442, 430
Notification : in Tuberculosis, 75 ; of Infectious
Diseases, Tin < A.,*, 145
Nutrition (P.1,153
Oath, Taking th"> (A..), 401
Open-air Treatment of Pnlmonarv Tuberculosis,
The Ultimate Results of (O.). 79
Operations, Gastric, History of Patients after (OA
405
Opkthatm'a, Same Forms of (P ). 246
Ophthalmology, Progi-ess in (P.), 63, 24S, 258
Opium Habit (P.), 118
Opportunities o' the Walthy, The. 219
Optologist and Pharmacist; (4.), 401
Overlaying of Infants, The (A.), 27
Pancreas and Spleen, Progress in Surgery of the
(Pt, 15
Pancreatic Disease (P.), 169
Paralysis: Br,wn-cequa'd's (P), 134 ; General,
of the insane (P.), 189, 191, 467; Gen?ral,
R?cent Views and Ob ervationa on (P.j, 116 ;
Ischtemic P.). 466 ; L?niry's (P.), 134
Pediatrics, Progress in CP.>, 447
Pensions to Asylum Offic a's W,), 281
Peiicaroium. Adherent P.) 101. (0)129
Peri'omy for Liffuse Corneiti- (P ), 66
Peritoneum. Clfansirg the (P.), f59; Sensibility of
the (P.), 2E9
Peritonitis Gonococcal (P.), 447.461; Tuberculous
(P), 101 155
Pertussis. 0 implication? and R'que'ae of (?.), 464
PQototh-rapy ; Bactericidal Fa vors in (P.), 189 ;
Physical Fact >rs in (P.), 183
PhotothoraDv and Rleccro-therapeuticJ, Progress
in (P ), 17- 33. 48
Plague (P.), 406 : The Mode of Infection by (i..)i
253 ; The pread of. by Flea' (0 ), 165
Pneumonia (P.), 118; Lobar, Pnysxal Signs of
(0.). 443
Poisons, Dispensing of (A..), 419; The Sale of (A..),
Ill
Post-Graduation Teachine in London 144
Practical Departments ( W ), 179. 284. 331. 471
Pregnancy, Tubal, Rt'obg / of (P ), 355
Property and Life (A..), 411
Prostate; Cancer of th ? (P.), 8'.; (0.), 115 ; San ile
Enlargement of the (P ) 82
Psychiatry, Progress in (P.), 101 116
Psychology of Faslion, t'h'(A.) 183
Psychopathy of Seers and Madiatjs (P.), 116
Public Health (A..) 335 ; The aad th 5 Duty of the
IJaily Pre^a 2: Mini'ter of. A., 91
Pupil; Argyll-Ribertson, Tie (P.), 423 ; Cbad'tion
of the, in Acute Alcoholic Poisoning (0 ) 2 Jo
Pyrexia in Cancar of the tiiver, 162
Qiackery and the Clergy (A.), 95
Radium Etnana'ions anl Rays (0 ), 257
Rainfall and Public Health (A.). 253
Reflexes; D agnostic ^igaiicaacj of Certain (P),
-463 ; Significance o' Sao r&c'a1 and Ten ioa, la
lower Extremity (0 ), 331; Tendon, in Uraemia
(P.), 465
Research and Post-G^da its tuly in \tadicin3, 53
Respiratory Diseases (P.) 102,113
Re-Vascination, 433
Rheumatic Children TheCara of (0.), 97
Rheimatic Iritis (A.) 43
Rheumalsm ; Acute (P ), 85 ; anl Gaut (P ), 425
Rhinitis, Atrophic (0 ), 273
Rickets, The Cause o' (0.), 431
Roman Catholic Inahriates aud their Religion, 26,
91
Royal Victoria Hospital, Belfast Tin (W.). 232
Royal Wat9rlio Hospital ior Worn in and Cailiran,
The ( W), 83 3D4
St. Bartholomew's H^snUal (W.), 158. 175, (A.)
201, (W.), 217, A.),269, (W.V 293 319
St. Rarthoi maws Hosp.tal and ttu Nation (W.),
?6
St. Bartho'omew's Hospital; The Crisis at '09;
The Future of (W. . 122 139,141,153 305
Salop Infirmirv, The (W >, 178
Sanity and Sieap (A.), 411
Sarcoma, Primary Retroparitoieii fPA 253
Sareomatosis, Multiple, of the Central Ner70us
Sjstem (P.), 50
3carlet Fever (P ). 237
Schmidt's (Dr. Ottj), Treatment of Cancer (0.),
113
Sciatica (P.) 426
Sclerosis, Amyotrophic Lateral (P.), 203
Scurvy. Infantile (P.), 100
Sepsis. Post- yphoid (C. . 13
Sawage, Pisp ^sil of London ( A ), 459
Snelifiah, Sewase Contamination o' (A.), 367
Snock, The Temperature 'n (O.l, 291
Sleeping Sickness (A.) Ill, (P.), 190,390
Small-pox (P ), 407
8ouihw?rk Pcandal, Not a Guy's bat a (A.), 339,
(W.), 431
Spine. Seme Points relat'ng to Rigidity of the (P.)
443
Spleen Rupture of the (PA 374
Standard sed Prep?rations A.) 349
State Medicine Progress in iP.) 63 83 134. 153
Stenosis ; Congenital Hypertrophic, of the P,lorua
(C >, 114 ; Pulmonary, c se simulatijg (P.),
393; of the Pyl >rns (P.). 32
Stimulants Cardiac H p viermic use of (0.), 423
Stoke- \dam Di=? ase t P. . 223
Stomach ; Dilatation of the (P ). 152 ; Progress in
Surgery of the PA 16 32. 276, 295
Student o' Medicine 24 40, 58, 71 92 108 121 112
160. 180,198,218 234 266, 284, 306, 326 ' 316!
361, 382 398 437 4b6. 472
Subco juiictival It j cti )ns P.), 258
Sundenand Dnion Infirmary ? *V ) 21
Surgery ; Abdomi- al (P). 259 ; Brain and Nerves
(P.), 316, 336; Cerebral, -pinal, and Nfr?e
(P.), 448 465; Gemto-Urinary (P). 82 ?95;
Intestinal (P.), 292; Liver, Gall-bladder a d
Spleen (.P.). 50 67 373; ?erve (P. , 34. 19;
Pancrtas and Spleen P.\ 15 ; Prog ess of (PA
168 293,315; Stomach P.), 16, 32. 276, 235 ;
Vermif >,m \poerdix (P. ,355
Snrgica! Aid Society, The (W.), i97
Swall .wing. D fflc llty in (C ). E8
Syph'litic A (Sections of the Heart anl Aorta (0.),
204
Syphilitic Joint Disease (C..\ 2Z6
J3Uirl lil?MKsi 1 TU J.iiU nU&ril AL, April
IV
Index to Vol. XXXV. THE HOSPITAL. 3rd October, 1903, to 23th March, 1904.
Tabes Dorsalis CP.). 191
Teeth in Inherited Syphilid The (A.), 237
Temperance Teaching in the Schools (A.), 27
Temperature. The, in Coma (A. ). 127
Temperatures Oral and Rental (P ), 103
"Terrible Imposture and Force of Worls," The,
125
^"stiDfr House for Reformed Drunkards ( V.\ 3
Tetanus Treated by Injections of A.nfcitocin into
the Spinal Thee* (C>, 445
Tonsils, Enlarged 0 ). 29
Torticillis, Spasmodic, relieved by Operation (P.),
50
Training Ships for Pauper Children (A.\ 419
Traumatic ^epiratioa of the Lower Epiphysis of
the Humerus (0.). 183
Treatment of tha Sic'* in Norwich during the
Rev nteenth Ceiturv. 172
Trees and Climate ( v.) 287
Trifid Stomach (P. ?. 295
Trop iic Changes, The Result of Nerve Inj iry (P.),
31
Tropical Medicine, University Diplomi in, 384
Trynanosoma (P.) 84
Trypwo omias*s(P.) 374
Tuberculin in Opathalmolos-y (P ). 258
Tuberculosis: (P). 63, 83, 103; Human and
Bovine (P.) 118,151: Psvcholosry of. 343
Tuberculous Olands in the Neck, Operative Treat-
ment of (0 ). 149
Tumoir. B-ain, Lirge Blond Cyst in Arashuoid
Spacf Simu'aMn? (P.), 336
Tumours, Brain (P.), 426 ; Relief of Pain in (P.),
426
Tutmarg, Oerehe'Ia-. and tlnir Treatment by
Op'r^tion (P ),
Typhoid Fever (P ), 274 ? Evrly Diagnosis of Per-
foration in rn.-), 290; in Tnfanc? aaiOh'ld-
hoid (0.), 47; Intesoinal Perforalisn in (P.)i
293
Ulce?. Gastric (P.), 276; After Hi3tory of (0.),
371
Ulners, Gastrin and Duod-mal (PA 16
" Unamployed " Proswioni (A.-). 201
TJ-b\n Trsatmeni of Sirgte&t Tuberculosis (H.), 12
Urfemia TrautniMc, Oured by Oystooomy (P.), 335
Ureteritis in the Female (P.), 82
Urinary Casta (0 ) 81
Urine; Blac't (0.). 4S2; The Value of Ureteral
OitheterisaSion and Oryoscopy of tha (0.),
313
Uterine Fibroids, Diagnosis and Treatment of
(P.). 338 : The Treatment of (0. >, 113
Uterus; the Operative Treatment of Retroveruon
and fteftexi'in of tha (OA 187 : Pos'erior Dis-
placement* of the (P.), 338; Rupture of the,
folioar ins; Ose*arian Section P.I,33?
Uterus and Adnexi, Tubarculosis of t he (P.), 338
Vaccinat'on Lw, Risj and Growth of (W.), 38,
103,323. 359
Vagina, Pibromyoma of ???he (P ), 355
Vnntilatioi of Railwiy 0irrUsres (P.), 103
Vermiform Appjudix, Progress of Sargery of
(P.), 355
"Vertebra, Recovery from Practura of Fifth
0 'rvical (P.), 443
Visits to Private Asylum* (W.), 2'?, 123, 193
Voluntary Roipitjls ana Paying Patleat3, 308
Volvu.ua (P.J, 291
Waut?d, Ten Thousand a Year (1.), 419
W?r, Mellcine aid, 335
Waafc we Owe to Exaarimin';? on Animals (0.\
1<35, 185, 203, 223, 242,255,271,239,311,331,
351
Will Londoi Awake ? CW \ 412
Wi33 and Enlightened Governors (&..), 127
Wom'n aad Bitting (A..), 221
Workhou e. The, and ttie Gaol (A..)- 95
Working Men and Hospital Management (A..), 77
Worm Infections (P.), 403
A'-Riys: (P.), 18; in Trachoma (P.), 66
THE SPECIAL SECTION FOR NURSES.
Ambidexterity in the Tnfant School, 325
American Society of Training Schools for Nurse3,
The. 68
Anglo-American Nursing Home, Rome, 131, 263,
273
An Impression from the Land of the 3hamrock, 29
Application of Hot Irrigation to a Leg with a
Compound Fracture, The, 232
Appointments
Association fo Promoting tha Training and Supp'y
of Mid wives, 152
Awakening of a Soul, The, 223
Bayond the Seas: NursioginaCawnpore Hospital
Book and its Story, A, 18, 49, 85,103,185, 237, 276,
303, 312
Bulbs for Hospital Decoration, 32
Central Midwives Board. 94, 272
Charing Crors Hospital, 15
Choosing thi New Matron of the Boyal Prince
Alfred Hospital. Sydney, 353
Christmas Books, 154, 167
Christmas in the Hopitals, 193, 205
Christmas in Forugn Hospitals?The Eppendorf
Hospitfil. Hamburcr. 141 ; The Rigsbospital,
Christian!a, 143; Berlin Municipal Hoepital,
145; Christmas and the Roman Hospita's,
147 ; Christmas at a Government Hospital in
Cyprus, 149
Conference on the Requirements of the Midwives
Act in Rural Dissrists, 218
Danger to Life from Burns and ScaMs, 337
Death in Our Ranks. 1?6,196 259, 289, 338
District Nursine and Diet, 164
Dot and Tiny, 179
East London Nursing Society, 339
Echoes from the Outside World
Everybody's Opinion
Evolution of the Modern Nurse, The, 1?0
Exam'nation of the Urine, The. 27 55
Exammatioa Questions for Nurje?, 16, 70, 92.164,
232, 287, 337
First Night iu a Private Nurses' Home, 42
For Klderly Nurses, 32
FunnMnn a', the North-Eastern Hospital for
Children, A, 233
Gynecological Nursing, 44
Hints on How t,o Start a District Nursing Associa-
tion, 8, 28. 43, 56
History of a Nursing Horn*. 129
Ho3oital and Private Nursing in Madeira, 311
How Nurses are Manufactured in Franca, 299
Incidents in the Pombav Fever Wards, 117
Infirmary Nurse Burnt to Death, 248
International Hoso'tal at Venice, The 31
Irish Dispensary Mid wive' and their Work, 42
Iiish Nurses' Association, The, 353
Lectures on Ophthalmic Nursing, 5, 41, 79, 103,
128,178, 204, 258, 284, 310
Lectures uoon the Nursin * of Mental Diseases,
115,163,192, 244. 270, 293, 322, 350
Locked in a Mortuary, 238
Lord Lytton on Districs Nursing, 339
Matfrnity Work in Pouth Africa: Nursing in a
Dutch Family 312
Miss Florence Nightingale and Belfast Nurses, 170
Modern Nursing of Consumption, The, 334
National Training School for District Midwives,
Tee, 338
National Union of Women Workers, 82
New Books on Nursing, 58. 95.118,181
Night Superintendent, The, 133
Notes on News from the Nursing Would
Nurses' Bookshelf, The, 34, 47, 219, 235,250, 278,
314
Nurses' M's ionarv Union, 153."
Nnreesof Bristol Genera' Boipita', The, 1C4
Nurses of Darlington Hospital, The, 165
Nurses of Leavesden Asylum for Imteci'cs, The,
285
Nunes of St. George's ia ton Bast, The, 130
Nui-s'b of St. Marylebone Infirmary, The, 215 *
Nuising bv Sisters of Meray, 6
Nursing Experience on Mount Lebanon, 4, 271
Nursing in Private Practice, Sir William Bennett
oil Modern, 216 233
Nursing in the Netherlands, 45
Nubsisg Outlook, The
Nursing under the Municipality in Paris, 11
Official Announcements 95
Our Onriatmas Didtributioj, 179
Preparation and AnnU^lon of an Abdominal
Fomentation, The, 287
Presentation 3
Pievent:on of Bed-Sores, Th*. 16, 70, 92
Private Nursing in Ruisia, 323
Queen Alexandra's Imperial Military Nursing
Service, 23?
Queen Victoria's Jubilee Institute for Nurse3, 203
Rojal British Nurses' Association, 253
St. Bartholomew's Hospital, Rochester, 233
Sketches at a Children's Hospital. 9
Some Practical Hints in the Nursing of Diseases
of the Skin, 67, 91
State Registration of Nurses, The, 218, 335, 351
Superintendent Nurse at Lady well Workhou3fr
and her Duties, The, 247
To Help the Nurses tn Help Themselves, 153
Training School for Nurses in Naples, A, 132
Travet, Noras and Queries
Victorian Trained Nurses' A:b jciaiion, The, 31
Workhouse Nursing;, 80, 93
Work of a Small Proviucial Hospital, Tha, 180
Printed by SP0TTI3WOODE & CO. Ltd., New Street Square, E.G.; and Published by the Proprietors,JTHE S3IENTIFI0 PRE3S, LIMITED,
at 28 & 29 Southampt.n Street, Strand, London, W.O.
THE HOSPITAL. Oct. 3r 1903,
NOTICE TO CORRESPONDENTS.
All UBS., Letters, Books for Review, and other matters intended for the
?dltor ihould be addressed The Editor, " The Hospital," The Hospital
Buildings, 28 & 29 Southampton Street, Strand, W.O.
The Editor cannot undertake to return rejected MS., even when
accompanied by stamped directed envelope.
Votlceilto Advertisers and Subscribers.
All Advertisements, Orders for Copies of The Hospital, and Business
Communications should be addressed to TBS MAVAGERt
(Wot to the Editor), TheoHospital, 28 & 29 Southampton Street,
Strand, London, W.O.
The Cost of Subscription, post free, Is as follows.?Per week, 3Jd.; per
quarter, 3s. 9d.; per half-year, 7s. 6d.; per annum, 15s. Foreign and
Colonial:?Per annum, 19s. 6d., payable, in all cases, in advance.
MOTXCE.?Vol. XXXXIX. of "The Hospital" (October
1902 to March 1903) In handsome cloth binding:,
will shortly be on sale at the office of this Journal,
price 7s. 6d. Cases for binding the Numbers
contained In this Volume Xs. 6d. Index to Vol.
3ESCZXXXI, 2}d., post free.
Vhe monthly part for October Is now ready.
Price Is., by post Is. 3d.
The Student of Medicine,
?aPPOXXTTMENTSt
T. Aubrey, M.B.Lond., M.R.C.S., District Medical Officer of
the Bridgwater Union. C. E. Bishop, M.R.C.S.Eng.,L.S.A., Dis-
trict Medical Officer of theShepton Mallet Union. J. B. Bowden,
M.D., M.S.Edin., Certifying Factory Surgeon for the Kidwelly
District, Carmarthenshire. Ernest Brice, L.R.C.P., Honorary
Life Member and Examiner to St. John Ambulanca Association,
j. "Walter Brown, B.A., M.B., B.Ch., Medical Officer of Health
for the County of Hokiargy, New Zealand, and Native Medical
Officer for the Hokiargy District. F. "W. Condon, F.R C.S.,
L.R.C.P.I., Certifying Factory Surgeon for the Ballyshannon Dis-
trict of the Counties of Donegal and Fermanagh. W. McAdam
Eccles, M.S.Lond., F.R.C.S.Eng., Joint Lecturer on Anatomy, St.
Bartholomew's Hospital. Walter Garstang, M.B., B.Ch/Vict.,
L.S.A.Lond., Honorary Surgeon to the Hulme Dispensary.
W. L. A. Leslie, M.B.Aberd., Assistant Medical Officer,
Grahamstown Asylum, South Africa. Leonard W. Light,,
M.R.C.S., L.R.C.P.Lond., Medical Officer and Public Vaccinator
for the Brad well District of the Maldon Union. "William C.
Millea, B.A., L.R.C.P.&S.Edin., Medical Officer of the South
District of the Poplar Union. It. H. Norman, M.D.Lond., B.S.r
M.R.C.S., L.R.C.P., Anaesthetist to the Tottenham Hospital. A.
Brown Ritchie, M.B., C.M.Edin, Consulting Surgeon to the
Hulme Dispensary. B. B. Thorn, M.B., Ch.B.Glasg., Certifying.
Factory Surgeon for the Linlithgow District of the County of
Linlithgow.
St. Bartholomew's Hospital.?The following appointments-
to the resident staff have been made : House Physicians.? Senior t
E. L. Martin, "W". W. Jeudwine, E. G. Pringle, P. H.
Woke, B. M. Banking. Junior: A. H. Hogarth, H. IT..
Gould, J. E. Payne, N. Macfadyen, C. B. V. Brown.
House Surgeons.? Senior : H. V. Wenham, V. G.Ward, A. M.
Amsler, H. Love, B. C. Elmslie. Junior: N. E. Waterfield,.
H. Statham, A. S. Petrie, T. J. Paulder, L. Noon. Resident
Midwifery Assistant.?E. E. Young. Extern Midwifery Assist-
ants.?E. L. Parncombe (October to January), A. P.
Alexander (January to April). Ophthalmic House Surgeon.?
B. B. Etherington-Smith.
" The Hospital" Institutional Library.
" Hospitals and Asylums of the World." 4 Vols., with a Port'
folio of Plans, complete ?12 12s.
" Burdett's Hospitals and Charities, 1903." 5s. net.
" Burdett's Official Nursing Directory, 1903." 3s. net.
" Cottage Hospitals, General, Fever, and Convalescent." 10s. 6d.
" Uniform System of Accounts for Hospitals and Public Institu-
tions." New Edition, 4s. net.
" Hospital Expenditure: The Commissariat." 2s. 6d.
"A Legal Handbook for the Use of Hospital Authorities.5*
2s. 6d.
"The Cottage Hospital Case Book or Register of Patients." 10&
and 12s. 6d.
All these are published by the Scientific Press, Ltd., and may
be obtained through any bookseller, or direct from the publishers,
28 and 29 Southampton Street, Strand, London, W.C.
2 Nursing Section. ?... THE HOSPITAL. Oct. 3, 1903.
NURSES' TEXT-BOOKS.
A HANDBOOK FOR NURSES.
By Dr. WATSON.
Price 5/- net, 5/4 post free.
This work is becoming more and more popular with the modern nurse, who finds
that nowadays she is required to know much that is not included in the older text-books.
Dr. Watson has succeeded in providing a complete guide to modern nursing.
SURGICAL WARD-WORK
AND NURSING.
By Dr. ALEXANDER MILES.
Price 3/6 net, 3/10 post free.
The Surgical Nurse will find this a most complete introduction to surgery and
surgical instruments. It is profusely illustrated.
THE HUMAN BODY.
By Professor BUEL COLTON.
Price 5/- post free.
We cannot too highly recommend this most interesting work on the Physiology,
Anatomy, and Hygiene of the Human Body. All three aspects are clearly and fully
explained, and much information connected with the subject is also given. It is not
merely a text-book; the intelligence and imagination of the student are constantly
appealed to, and useful hints are also given to teachers.
THE ART OF MASSAGE.
By A. CREIGHTON HALE.
Price 6/- post free.
In this work Mrs. Creighton Hale has embodied her vast experience as a teacher,
and has made her work complete by numerous and excellent illustrations.
THE NURSES' DICTIONARY
OF MEDICAL TERMS.
By HONNOR MORTEN.
Price 2/- post free.
This is an invaluable companion for every nurse.
OPHTHALMIC NURSING.
By SYDNEY STEPHENSON, F.R.C.S.E.
Price 3/6 net, 3/10 post free.
An admirable guide to the nursing of eye cases.
These books are obtainable of all Booksellers, and are published by
THE SCIENTIFIC PRESS, LIMITED, 28 & 29 Southampton Street, Strand, London,
who will send their latest Catalogue of Nursing Text-books post free to any Nurse
on application.
lx Nursing Section. THE HOSPITAL. Oct. 3, 1903.
WORKS FOR NURSES.
LESSONS ON MASSAGE. By Mrs.
MARGARET D. PALMER, Manager of the Massage
Department and Instructor of Massage to the Nursing
StafE of the London Hospital. Second Edition enlarged
and revised; pp. xvi. + 262 with 118 Illustrations,
plain and coloured. Just Published. Price 7/6 net.
MANUAL FOR STUDENTS OF
MASSAGE. By Miss M. A. ELLISON, L.O.S. With
50 original Illustrations. Price 3/6 net.
A MANUAL FOR HOME NURSING.
By BERNARD MYERS, M.D., M.R C.S., Surgeon and
Lecturer to St. John's Ambulanca Association. Just
Published. Price 2/6 net.
NOTES ON HOME NURSING. By Miss
D. M. GOLDIE, Lecturer on Nursing, Hygiene, &c., to
the County Councils of London, Surrey and Middlesex.
Just Published. Price 1/6 net.
THE FEEDING AND HYGIENE OF
INFANTS AND CHILDREN. By JOHN
McCAW, M.D,, M.R.C.P., Pdjfeician to the Belfast
Hospital for Children. Just Published. Price 2/6.
DISEASES OFH"HE HAIR, INCLUD-
ING GREYNESS, BALDNESS, &c., AND
THEIR TREATMENT. By DAVIL) vVALSH,
M.D., Western Hospital for Diseases of the Skin.
Price 2/6 1 et.
MENSTRUATION AND ITS DIS-
ORDERS. By ARTHUR E. GILES M.D., B.Sc.,
F.R.C.S, Surgeon Chelsea Hospital for Women. Price
2/6 net.
MOVABLE ATLAS OF THE
ANATOMY AND PHYSIOLOGY OF THE
FEMALE GENERATIVE ORGANS AND OF
PREGNANCY. Second Edition. With Test. By
ARTHUR E. GILES, M.D., F.R.C.S. Price 3/-net.
MOVABLE ATLAS OF THE
ANATOMY AND PHYSIOLOGY OF THE
CHILD. With Explanatory Text. By D'ARCY
POWER, M.A.Oxon, M.B., F.R.C.S. Price 3/- net.
BAILLIERE'S POPULAR MOVABLE
ATLAS OF THE ANATOMY AND PHYSI-
OLOGY OF MAN. Size 16 by 7| inches. With
Explanatory Text. By W. S. FURNEAUX, Special
Science Teacher London School Board. Price 3/6 net.
London : BAILLIERE, TINDALL & COX, 8 Henrietta Street, Covent Garden.
Oct. 3, 1903.  THE HOSPITAL. Nursing Section, lxi
NOW HEADY,
^ ? REPRINT OP
A Nursing handbook
TOGETHER WITH B. MAW, SON AND SONS'
GOMPLETE CATALOGUE OF NURSING REQUISITES.
The above work is now republished at a cost of Is. per copy. Messrs. S. M.,
S. and S. have been compelled to make this charge on account of the extreme
popularity of the work and the great expense incurred in its production,
Post Free on receipt of Is,
\ S. MAW, SON and SONS, ^
\ 1 to 12 ALDERSGATE STREET, LONDON, E.C,

				

